# Supporting Simultaneous Air Revitalization and Thermal Control in a Crewed Habitat With Temperate *Chlorella vulgaris* and Eurythermic Antarctic Chlorophyta

**DOI:** 10.3389/fmicb.2021.709746

**Published:** 2021-08-24

**Authors:** Emily E. Matula, James A. Nabity, Diane M. McKnight

**Affiliations:** ^1^Bioastronautics, Smead Aerospace Engineering Sciences, University of Colorado Boulder, Boulder, CO, United States; ^2^Institute of Arctic and Alpine Research, University of Colorado Boulder, Boulder, CO, United States; ^3^Department of Civil, Environmental and Architectural Engineering, University of Colorado Boulder, Boulder, CO, United States

**Keywords:** bioregenerative life support systems, Antarctica, McMurdo Dry Valleys, thermal control, air revitalization, Chlorophyta

## Abstract

Including a multifunctional, bioregenerative algal photobioreactor for simultaneous air revitalization and thermal control may aid in carbon loop closure for long-duration surface habitats. However, using water-based algal media as a cabin heat sink may expose the contained culture to a dynamic, low temperature environment. Including psychrotolerant microalgae, native to these temperature regimes, in the photobioreactor may contribute to system stability. This paper assesses the impact of a cycled temperature environment, reflective of spacecraft thermal loops, to the oxygen provision capability of temperate *Chlorella vulgaris* and eurythermic Antarctic Chlorophyta. The tested 28-min temperature cycles reflected the internal thermal control loops of the International Space Station (*C*. *vulgaris*, 9–27°C; Chlorophyta-Ant, 4–14°C) and included a constant temperature control (10°C). Both sample types of the cycled temperature condition concluded with increased oxygen production rates (*C*. *vulgaris*; initial: 0.013 mgO_2_ L^–1^, final: 3.15 mgO_2_ L^–1^ and Chlorophyta-Ant; initial: 0.653 mgO_2_ L^–1^, final: 1.03 mgO_2_ L^–1^) and culture growth, suggesting environmental acclimation. Antarctic sample conditions exhibited increases or sustainment of oxygen production rates normalized by biomass dry weight, while both *C*. *vulgaris* sample conditions decreased oxygen production per biomass. However, even with the temperature-induced reduction, cycled temperature *C*. *vulgaris* had a significantly higher normalized oxygen production rate than Antarctic Chlorophyta. Chlorophyll fluorometry measurements showed that the cycled temperature conditions did not overly stress both sample types (F_V_/F_M_: 0.6–0.75), but the Antarctic Chlorophyta sample had significantly higher fluorometry readings than its *C*. *vulgaris* counterpart (*F* = 6.26, *P* < 0.05). The steady state *C*. *vulgaris* condition had significantly lower fluorometry readings than all other conditions (F_V_/F_M_: 0.34), suggesting a stressed culture. This study compares the results to similar experiments conducted in steady state or diurnally cycled temperature conditions. Recommendations for surface system implementation are based off the presented results. The preliminary findings imply that both *C*. *vulgaris* and Antarctic Chlorophyta can withstand the dynamic temperature environment reflective of a thermal control loop and these data can be used for future design models.

## Introduction

Engineers have developed technologies to provide air revitalization [carbon dioxide (CO_2_) scrubbing/oxygen (O_2_) provision], waste removal and processing, food, and thermal control for crewed spaceflight. While these systems have been proven on the International Space Station (ISS) and in Low-Earth Orbit (LEO), many require regular and frequent resupply missions (NASA, 2015). With the current launch technologies, this approach is unsustainable for longer duration missions traveling farther from Earth, as resupply missions can become mass prohibitive (NASA, 2015). Therefore, exploring closed-loop, robust, and adaptable environmental control and life support (ECLS) systems for longer duration and surface missions (i.e., Martian and Lunar missions) is imperative. Using multifunctional approaches that address multiple life support roles concurrently may further reduce system mass, power, and volume (Matula and Nabity, 2016).

Bioregenerative ECLS technologies, specifically algal photobioreactors, can simultaneously fix atmospheric carbon dioxide and produce oxygen through photosynthesis. Additional species-dependent functionalities include waste remediation (through nitrogen and phosphorous assimilation) and producing edible biomass (thereby providing carbon and nutrient loop closure) (Chinnasamy et al., 2009; Kumar et al., 2010; Jaatinen et al., 2015; Tuantet et al., 2019). Preliminary studies using photobioreactors for the support of human spaceflight were conducted both terrestrially and in LEO (Popova et al., 1989; Gilles et al., 2008; Lasseur et al., 2011; Helisch et al., 2020; Poughon et al., 2020). Typically, these systems focused on food production and air revitalization (Javanmardian and Palsson, 1991; Li et al., 2013).

**FIGURE 1:**
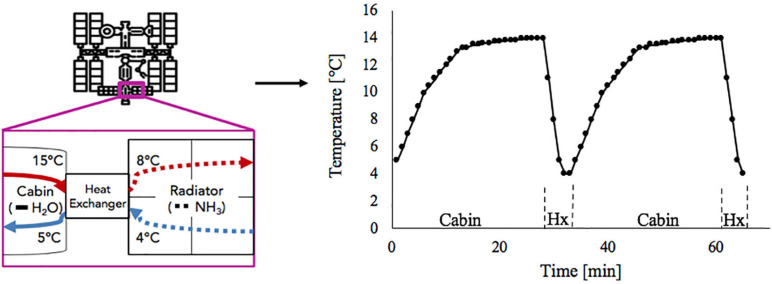
Absorption of cabin heat and heat exchange between low temperature loop and ammonia radiator (*left*) results in temperature cycles in low temperature loop water as it flows between cabin and heat exchanger (*right*).

Including the water-based growth media in the culture utilization design space can offer added benefit to a multifunctional system. The current ISS thermal control system and proposed sustained surface missions use water loops snaked throughout the cabin walls to absorb cabin waste heat before radiating it off to space (Reysa and Thurman, 1997; Westheimer, 2019; NASA, 2020). Taking advantage of the already allocated cabin volume and plumbing, using algal media in place of water for thermal control may further reduce ECLS mass, power, and volume (Matula and Nabity, 2016). However, using media for thermal control could expose the algal culture to a very dynamic thermal environment (Figure 1). This temperature profile depends on the commanded flow rate and cabin heat load. Table 1 presents the operating characteristics of each of the ISS internal thermal loops and spacecraft cabin (The Boeing Company, 2001; Valenzano et al., 2003; Anderson et al., 2018). Mass production of microalgae in raceway ponds may sustain diel temperature cycles from +10 to +45°C (Ras et al., 2013). However, many studies have shown that, within a species-dependent range, there is a positive correlation of photosynthetic rate to temperature (Öquist, 1983; Dauta et al., 1990; Davidson, 1991; Ras et al., 2013). Therefore, a reduction in environmental temperature may lead to a decrease in the air revitalization capacity of these photobioreactors (Maxwell et al., 1994; Morales et al., 2018).

**TABLE 1:** Internal active thermal control system and crewed cabin operational characteristics (The Boeing Company, 2001; Valenzano et al., 2003; Anderson et al., 2018).

Internal system	Operational temperature range (°C)	Loop volume (L)	Heat transferred (kW)
Lower temperature loop (LTL)	3.0–15	63	7
Moderate temperature loop (MTL)	10–21	200	12.5
Spacecraft cabin	18–27	–	–

Maxwell et al. successfully sustained *Chlorella vulgaris* at a constant +5 and +27°C for 10 days. They noted the +5°C culture reduced cellular chlorophyll (Chl) content; thereby preserving cell sustainment through reduced irradiance absorption and excitation pressure on photosystem II (PSII) (Maxwell et al., 1994; Morgan-Kiss et al., 2006; Teoh et al., 2013). Limited studies investigating the effects of diurnal cycles in irradiance and temperature (+3 to +28°C) suggest that microalgae are adaptable and viable beyond optimal culturing conditions (+26 to +36°C) (Chisti, 2007; Ugwu et al., 2007; Costache et al., 2013). Additionally, there was no significant difference in specific growth rate between cultures experiencing diurnal cycling and time-averaged constant temperature controls (Tamiya et al., 1955; Tamburic et al., 2014; Yang et al., 2016). Davidson suggested that microalgae easily acclimate to temperature change over the course of weeks to days. He stated, however, that little is known about the rate of acclimation, especially within 24-h time scales; which a brief literature survey showed is still true today (Davidson, 1991). Spacecraft thermal loops can have a turnover rate of a few minutes. References for this study reported the optimal temperature range for *Chlorella* taxa was just within the spacecraft cabin temperature range (Table 1; Dauta et al., 1990; Maxwell et al., 1994; Serra-Maia et al., 2016; Anderson et al., 2018).

The referenced studies were conducted with lab-sustained *Chlorella* taxa, a readily available freshwater genus that easily grows in a range of temperature and pH conditions (Mayo, 1997; Ras et al., 2013; Zhao et al., 2013). Non-toxic to humans, and after minimal processing, it is fit for consumption as nutritional supplementation, closing the carbon loop for spaceflight (Powell et al., 1961; Hedenskog et al., 1969; Gitelson et al., 2003; Morris et al., 2008; Carlson, 2011; Solter and Beasley, 2013; Tibbetts et al., 2015). Therefore, this genus is widely accepted by the spaceflight community and has extensively published terrestrial data with limited spaceflight studies (Myers and Johnston, 1948, 1949; Myers, 1964; Lasseur et al., 2011; Drexler and Yeh, 2014; Serra-Maia et al., 2016; Rabbow et al., 2017; Niederwieser et al., 2018; de Vera et al., 2019; Helisch et al., 2020; Pickett et al., 2020).

Ras et al. (2013) denoted that microalgae generally adapt to a new environment over the course of a season, acclimating to the environment after enough generations. They added, “Optimal temperature should therefore be associated to the environmental conditions for which they (microalgae) have been obtained.” This implies that psychrotolerant or cryophilic microalgae may be the most appropriate for use in a thermal control system, as they may be tolerant of the temperature extremes found in a cabin cooling loop. Spaceflight designs may benefit from bioprospecting by using extremophiles, taking advantage of their robustness and adapted metabolic processes (Beattie et al., 2011; Malavasi et al., 2020).

The McMurdo Dry Valleys (MDV), located on the west coast of McMurdo Sound, is the largest polar desert in Antarctica and exceptional planetary (Martian) analog (Doran et al., 2010). The MDV is home to ephemeral, glacial-fed streams where diverse types of perennial algal mats are common. These extremophilic microalgae grow as dense mats consisting of mixtures of cyanobacteria and eukaryotic microalgae (including taxa of *Prasiola*, *Chlorella*, and *Chlamydomonas*) (Kohler et al., 2016; Van Horn et al., 2016). Desiccated and cryopreserved in 24-h darkness for the winter, these mats photosynthesize within 10–20 min of rewetting in the summer, when the first glacial melt water saturates the stream bed and surface flow begins (McKnight et al., 1999; Darling et al., 2017). Through the summer, these species experience daily thermal swings between +2 and +15°C. This temperature range is driven by changing solar positions, where higher temperatures correlate to greater irradiance over the course of the summer. In the MDV, the 24-h irradiance in the summer imparts four times more UV-radiation than occurs in the United States (Cozzetto et al., 2006; Darling et al., 2017; Obryk et al., 2018; Sengupta et al., 2018). The irradiance conditions, along with the nutrients mobilized from weathering reactions in the underlying sediment of the streambed, supports this cold-environment photosynthesis. The freshwater algal mats from this location thrive in an environment analogous to conditions within a planetary habitat (Gooseff et al., 2010).

This study compares the air revitalization capabilities of commercially grown and Antarctic-sampled Chlorophyta, as potential microalgae for use in a multifunctional bioregenerative life support system. The thermal cycles imparted on these microalgae during the experiment are reflective of a spacecraft or surface habitat thermal loop. The work presented in this paper is the first comparative study investigating the impact of rapid, dynamic thermal environment on psychrotolerant and temperate-grown microalgae.

## Materials and Methods

### Site Description and Sample Methods

This study included *Chlorella vulgaris* (Bacteria-free agar slant, item #152075 isolated from UTEX 398, Carolina Biological) and green (Chlorophyta-Ant) mat gathered from the McMurdo Dry Valley, Antarctica.

**FIGURE 2:**
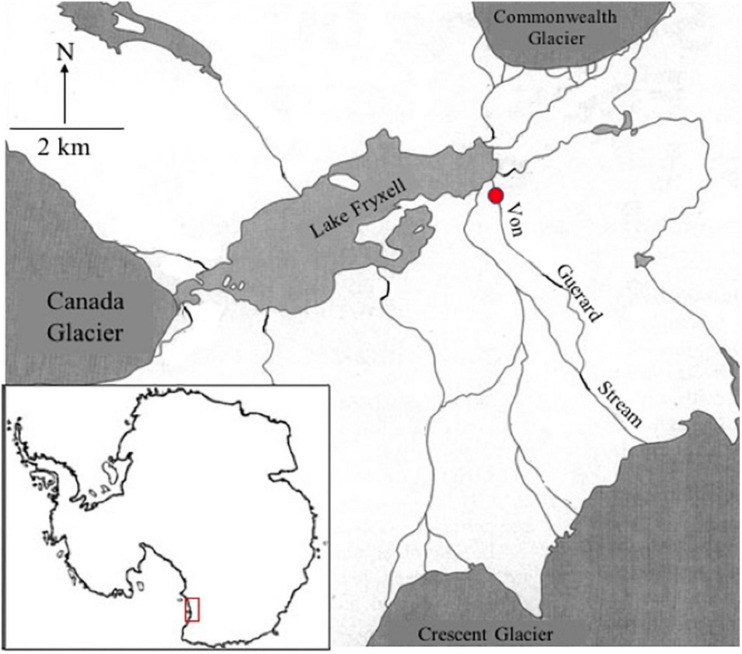
Reference map of the sampled McMurdo Dry Valleys (MDV), Antarctica (red box, *lower left*). Sample site a Von Guerard Stream is marked with a red circle.

Green algal mat was collected from Von Guerard stream in the Taylor Valley (77°26′38.717″S, 163°9′33.321″E) of the McMurdo Dry Valley, South Victoria Land, Antarctica (Figure 2). Sampling was conducted in January 2019 from the main thalweg of the stream ensuring, peak stream flow. Von Guerard Stream drains off of Von Guerard Glacier in the Kukri Hills, east of the Crescent Glacier (Cozzetto et al., 2006). Preliminary analysis of these green mats by Van Horn et al. (2016) suggested they are comprised of taxa of the genera *Prasiola*, *Chlorococcum*, and *Chlorella*. The availability of these species was confirmed through both light microscope (Olympus IMT, 40× dry objective) and fluid imagery microcopy (FlowCAM VS-IV, 10×).

Forceps and a spatula, triple-rinsed in stream water, were used to gather 3 cm × 6 cm sections of mat. Mat sections were each placed in a sterile 200 mL Nalgene screw-top sample bottles, triple-rinsed in stream water before sample deposit, and filled with approximately 100 mL of associated stream water. At the University of Colorado, the lids to the bottles were loosened to allow for ventilation and the foil-wrapped bottles were placed on a dark shelf of a +7°C (±1°C) incubator.

### Culturing Conditions

Subsamples of mat were transferred from the sample bottles to 250 mL Erlenmeyer flasks of 125 mL sterile Bold’s (Bristol’s) Modified Media (50× Bold Modified Basal Freshwater Nutrient Solution, Sigma Aldrich) and lightly capped with foil. After final dilution with ultrapure water (Milli-Q Direct, Millipore-Sigma), the freshwater media contained 2.94 mM NaNO_3_, 0.17 mM CaCl_2_–H_2_O, 0.3 mM mgSO_2_–7H_2_O, 0.43 mM K_2_HPO_4_, 1.29 mM KH_2_PO_4_, and 0.43 mM NaCl (pH 6.4). Over the incubation period, sterile Bold’s media was added to the flasks to return the total volume to 125 mL. The flasks were placed on an orbital shaker table, in the +7°C incubator, under a cool fluorescent lamp providing an irradiance of 50 μmol m^–1^ s^–1^ with a 12:12-h light:dark cycle. The incubator temperature was reflective of Von Guerard’s average summer temperature (+7°C) (Cozzetto et al., 2006). While the Antarctic austral summer has 24-h sunlight, the light:dark cycles reflect the relative motion of the sun across the MDV, which produces diurnal cycles of direct solar radiation on the microbial mats (Darling et al., 2017).

A continuous, xenic seed culture of *C*. *vulgaris* was sustained at a constant 20°C in Bold’s media. The 1 L Pyrex beaker of maintained mother culture was placed on a magnetic stir plate, under a bank of fluorescent bulbs (F40PL/AQ/ECO 49893 Bulb, GE Lighting) providing 24-h irradiance. The top of the beaker was covered lightly with clear cling wrap to allow 120 μmol m^–1^ s^–1^ irradiance at the culture surface. Free gas exchange with the clean, but not sterile, lab environment more closely replicated direct exposure to spacecraft or surface habitat conditions.

### Temperature Treatment

A Peltier cooler system was developed to control the temperature of the 12-well plates (229111, CellTreat) used in these experiments. The thermal loop profile was replicated using an Arduino-based system (Mega, Arduino). The Arduino received temperature input through a K-type thermocouple (K-type braided, Omega and K-type Thermocouple Amplifier, AdaFruit), and controlled the Peltier cooler (Peltier Thermo-Electric Cooler, Adafruit), in conjunction with a resistive heating pad (Heating Pad, Sparkfun). Those wells in direct contact with the cooler (B2, B3, and B4) were filled with 3.5 mL of culture. The initial culture density of *C*. *vulgaris* was 5 × 10^5^ cell mL^–1^ and Chlorophyta-Ant was approximately 0.34 mg mL^–1^. The plates had free gas exchange through a clear, gas-permeable membrane (Sealing membrane, Breathe-Easy) that sealed the tops of the plates to reduce evaporation. The membrane created a seal around the inserted measurement probes (pH, dissolved oxygen, temperature). An orbital shaker table (7744-01000, Bellco Orbital Shaker) continuously agitated the plates at 100 RPM, to reduce media temperature gradients within the well and promote gas exchange between the environment and media. Overhead lighting (F40PL/AQ/ECO 49893 Bulb, GE Lighting) provided 120 μmol m^–1^ s^–1^ at the plate surface, under 24-h irradiance. Dauta et al. (1990) suggests that this irradiance intensity reduced the risk of photoinhibition in *C*. *vulgaris* for the tested temperatures (Morales et al., 2018). This system was at pressure equilibrium with the surrounding environment in Boulder, CO (approximately 84.1 kPa). Triplicate experiments were executed for 7 days to capture the exponential growth phase.

**FIGURE 3:**
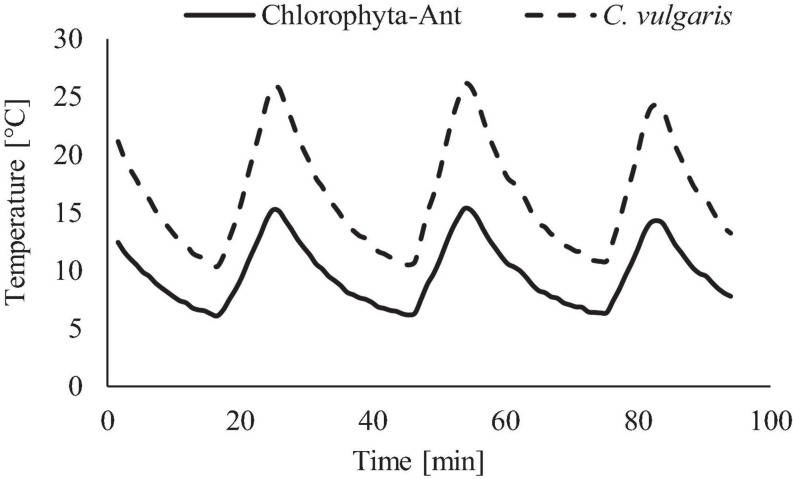
Temperature profile used for Chlorophyta-Ant and *C*. *vulgaris*, representative of spacecraft thermal loops.

The range and period of the tested sinusoidal temperature cycles were modeled from the ISS’s thermal control system and cabin environment (Figure 3). Temperature ranges for each experiment were adjusted to span both the operational conditions of the thermal loop but also the in-situ sample temperatures of each sample. The *C*. *vulgaris* experiment used a +9 to +27°C (±2°C) temperature profile, which included the LTL and cabin environment (Anderson et al., 2018). The Chlorophyta-Ant experiment used +4 to +14°C (±2°C), which replicated the operational range of the LTL [personal communication, Michael Holt, NASA Crew Thermal Systems, May 18, 2017]. The lower temperature bounds were dictated by the cooling capacity of the Peltier cooler and environmental chamber. The 28 min cycle period reflected the flow rate of the ISS Internal Active Thermal Control System (IATCS) (Hanford and Ewert, 1996; Patel et al., 2001; The Boeing Company, 2001). A constant temperature control was executed for both sample types at 10°C, which was the time-averaged temperature of the Chlorophyta-Ant experiment but also within the *Chlorella* experiment temperature range.

### Real-Time Measurements

PLX-DAQ software continuously recorded well temperature and pH (pH kit, Atlas-Scientific) measurements. Dissolved oxygen (Robust Oxygen Probe, PyroScience), with associated temperature compensation, was measured and recorded with the associated PyroScience software. All measurements had a sampling rate of 0.2 Hz. Before inoculating with algal culture, an operational baseline for dissolved oxygen and pH was established with sterile Bold’s media using the tested temperature profiles for 3 days.

### Non-continuous Measurements

Invasive sampling methods are required for calculating specific growth rate, and a minimal amount of biomass was available for each sample type. Therefore, the biomass measurement frequency was restricted to the start of end of each experimental run. *C*. *vulgaris* experiments used optical density measurements to measure biomass growth via spectrophotometer (Thermo Scientific Multiskan FC, Fisher Scientific) at 690 nm (Raso et al., 2011; Santos-Ballardo et al., 2015). The equivalent biomass dry weight was calculated using a conversion constant calculated from optical density (OD) measurements of standard dilutions of inoculate and corresponding dry weight biomass density measurements.

(1) $$Dryweight\left[gL^{-1}\right]=1.46\times OD_{690}$$

Both Chlorophyta-Ant experiments and the optical density conversion (Eq. 1) used a vacuum filtration method for preparing samples for biomass dry weight measurements. Well samples were deposited on quartz fiber filters (25 mm QE200, Advantec) and triple rinsed with ultrapure water before drying in a 60°C oven for >24-h.

Daily measurements of chlorophyll fluorometry provided a qualitative assessment of the cell’s photosystem II (PSII) response efficiency (measured on a scale of 0–1, 1 being the highest efficiency). This non-invasive method determined relative culture health, referred to as photosynthetic quantum yield [Y(II)]. Measuring a well containing 3.5 mL of sterile Bristol’s media before each culture measurement calibrated the pulse amplitude modulation system (Junior-PAM, Walz). Cultures were allowed to come to a steady state temperature (19°C for *C*. *vulgaris*, 10°C for Chlorophyta-Ant) after turning off the overhead light panel for at least 15 min to measure maximum fluorescence (F_V_/F_M_) (Schreiber, 2004). The PAM probe was held to the bottom of the culturing plate, in consistent locations, and the saturation pulse was initiated and recorded with associated PAM-Walz software (saturation: 1,500 μmol m^–1^ s^–1^, 0.4 s; actinic light: 420 μmol m^–1^ s^–1^).

### Analytical Methods

Dissolved oxygen, pH, and PAM measurements were recorded in 24-h increments and were post-processed with the corresponding Bold’s baseline.

(2) $$\Delta O_{2}=O_{2t}-O_{2sat}^{*}$$

Where Δ*O*_2_ is the excess dissolved oxygen concentration, g L^–1^, *O*_2_*_*t*_* is the measured dissolved oxygen concentration, g L^–1^, and *O^∗^_2sat_* is expected oxygen saturation for the media at the measured temperature, g L^–1^. Real-time data (pH and dissolved oxygen) were averaged over the 24-h period for both the control and cycled temperature conditions.

While it was understood that both sample types would experience a lag phase, the onset of exponential growth using biomass was not identified due to the restricted biomass sampling schedule. Therefore, the specific growth rate could not be calculated, and the biomass yield rate was used instead. The yield rate was calculated with the biomass dry weight and the following equation

(3) $$Y=\left({\text{N}}_{\text{t}}-N_{t0}\right)/\left(t-t_{0}\right)$$

Where *Y* is the biomass yield rate, gDW L^–1^ d^–1^, *N*(*t* or *t*_0_) is the biomass dry weight at time *t* and *t*_0_, gDW L^–1^, and *t* is time, *d*. The yield rate was used to compare biomass accumulation rates over the course of the experiment (Wood et al., 2005). Resulting media pH, oxygen production, yield rates, and photosynthetic yield measurements were averaged across the triplicate experiments.

Two-way ANOVA with replication determined statistical significance of real-time measurements between sample types with subsequent Tukey *post hoc* tests. One-way ANOVA calculated the significance of data associated with biomass dry weight. All statistical calculations were conducted with Microsoft Excel 2016 with the Data Analysis package with a critical value of *P* < 0.05 for all statistical tests. All presented data represents averaged triplicate results, and the associated standard deviation as the error bars calculated by Microsoft Excel 2016, unless specifically noted.

## Results

### Biomass Yield Rate

The clumping nature of Chlorophyta-Ant made it difficult to prepare consistent, homogeneous samples for daily measurements. Therefore, biomass dry weight was measured at the beginning and end of each experiment. Table 2 presents the calculated (Eq. 3) yield rate for each experiment. The temperature-cycled *C*. *vulgaris* condition had a significantly higher yield rate than the Chlorophyta-Ant sample (*F* = 9.44, *P* < 0.05); while the yield rate under steady-state temperatures (10°C) were similar magnitude for both sample types (*F* = 0.02, *P* > 0.05). The growth rate of Chlorophyta-Ant cultured at a steady state temperature was comparable to the cycled temperature condition (*F* = 0.01, *P* > 0.05). However, the yield rates for this sample type were less than those reported in literature culturing within similar temperature environments (0.11–0.15 g L^–1^ d^–1^) (Yang et al., 2016). The yield rate of the *C*. *vulgaris* sample type at a constant temperature was also less than reported values (0.14–0.17 g L^–1^ d^–1^). Yang et al. (2016) subjected isolated, temperate *Chlorella* to diurnal temperature fluctuations between 10°C/28°C, this condition resulted in growth rates significantly less than that presented here (0.21 g L^–1^ d^–1^).

**TABLE 2:** Biomass yield rates calculated from biomass dry weight measurements (Chlorophyta-Ant) and OD_690_ conversion (*Chlorella vulgaris*).

Experimental condition	Y (gDW L^–1^ day^–1^)
Chlorophyta-Ant (4–14°C)	0.02 ± 0.03^A^
*C*. *vulgaris* (9–27°C)	0.75 ± 0.41^B^
Chlorophyta-Ant (10°C)	0.02 ± 0.04^A^
*C*. *vulgaris* (10°C)	0.03 ± 0.02^A^

*Different letters in the superscript next to each value indicate significant differences (*P* < 0.05) between temperature conditions.*

### Oxygen Production

**FIGURE 4:**
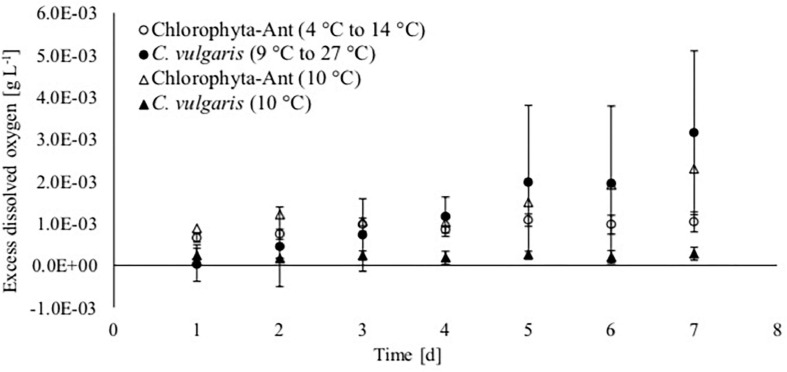
Excess dissolved oxygen based on saturated dissolved oxygen measurements of Antarctic Chlorophyta and temperate *Chlorella vulgaris* grown in thermal environments of a spacecraft.

Oxygen production increased over the course of the experiment for each treatment and sample type, except for *C*. *vulgaris* at the steady state 10°C condition (Figure 4). The dissolved oxygen probe was available for only one of the Chlorophyta-Ant at 10°C trials. Therefore, these preliminary data do not have associated standard deviation and they are presented for the comparisons of trends. The isolated trial for Chlorophyta-Ant under constant temperature conditions resulted in an 160% increase in dissolved oxygen. Both temperature-cycled conditions (Chlorophyta-Ant and *C*. *vulgaris*) had significantly higher dissolved oxygen than the constant 10°C *C*. *vulgaris* trials (*F* = 205.9, *P* < 0.001; *F* = 14.7, *P* < 0.001, respectively). While the temperature-cycled *Chlorella* condition experiments concluded with an excess dissolved oxygen concentration three times greater than that of the Chlorophyta-Ant sample type (3.15 versus 1.03 mgO_2_ L^–1^), the difference was insignificant (*F* = 2.28, *P* > 0.05).

Excess dissolved oxygen measurements were normalized with corresponding biomass dry weight measurements, as it is indicative of algal response to the culturing environment (Table 3). The *C*. *vulgaris* sample types decreased in normalized dissolved oxygen (9–27°C: decreased by 37%; 10°C: decreased by 22%). The normalized dissolved oxygen for the cycled temperature *C*. *vulgaris* was significantly reduced over the course of the experiment (*t* = 4.3, *P* < 0.05). However, the cycled temperature *C*. *vulgaris* results were significantly greater than both the cycled Chlorophyta-Ant and the constant temperature *C*. *vulgaris* condition (Chlorophyta Ant: *F* = 2.91, *P* < 0.05; constant *C*. *vulgaris*: *F* = 7.85, *P* < 0.05). The Chlorophyta-Ant (9–27°C) sample type rate increased by 33%. The constant temperature Chlorophyta-Ant resulted in a slight reduction in normalized dissolved oxygen concentration. Only one constant temperature Chlorophyta-Ant experiment had dissolved oxygen readings, therefore additional trial runs are needed to confirm the precision of this observation.

**TABLE 3:** Excess dissolved oxygen normalized with corresponding measured biomass dry weight.

Experimental condition	Initial (mgO_2_ gDW^–1^)	Final (mgO_2_ gDW^–1^)
Chlorophyta-Ant (4–14°C)	2.24 ± 0.42^A^	3.0 ± 1.71^A^
*C*. *vulgaris* (9–27°C)	8.84 ± 4.40^B^	6.51 ± 1.88^C^
Chlorophyta-Ant (10°C)	2.57	2.34
*C*. *vulgaris* (10°C)	4.45 ± 3.89^A^	3.47 ± 1.26^A^

*Superscripts indicate significant difference between initial and final values and within test conditions (P < 0.05).*

### Media pH

**FIGURE 5:**
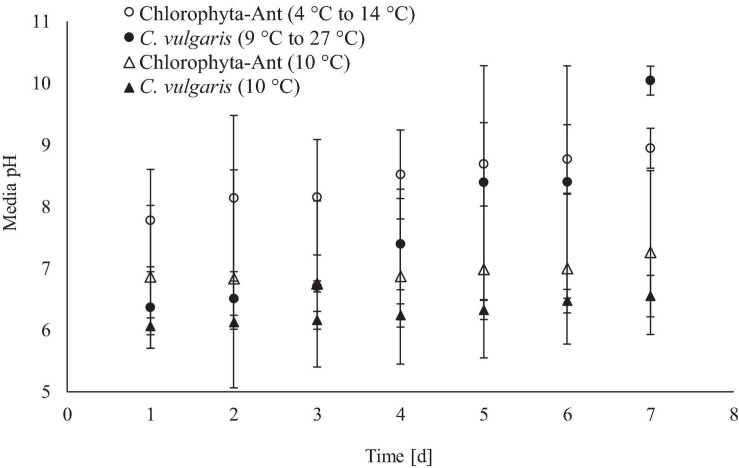
Real-time media pH measurements averaged across 24 h for qualitative insight of culture growth.

The increase in media pH through the execution of this experiment was expected for both sample types across all test conditions, as cultures fixed dissolved carbon species (Figure 5). The cycled temperature condition of the *C*. *vulgaris* sample type was the only case to result in a significant increase in media pH (Initial pH: 6.4, Final pH: 10.0; *F* = 5.14, *P* < 0.01). While both *C*. *vulgaris* temperature conditions started at approximately the same media pH, the cycled temperature condition had a significantly greater increase in pH than the constant temperature condition (*F* = 24.31, *P* < 0.001).

### Chlorophyll Fluorescence

Daily maximum chlorophyll fluorescence measurements were used as both a proxy for culture stress levels and estimating the onset of exponential growth. Both *C*. *vulgaris* conditions experienced at least a 2-day lag phase. The significant decrease in F_V_/F_M_ values, in conjunction with a decrease in pH and oxygen production, suggested the lag phase duration. As cultures acclimated to the culturing environment, a subsequent rise in values followed (Maxwell and Giles, 2000; Kula et al., 2017). The F_V_/F_M_ values for all conditions peaked around day six before declining again on day seven. It was hypothesized this was the onset of the stationary phase. As the Chlorophyta-Ant mats were partially comprised of *Chlorella*, it was assumed that the Chlorophyta-Ant sample type exhibited the same F_V_/F_M_ patterns as *C*. *vulgaris* while transitioning into the various growth regimes. The constant temperature *C*. *vulgaris* condition had significantly lower fluorescence values than all other tested conditions (*F* = 47.55, *P* < 0.001). Although all tested conditions resulted in reduced maximum fluorescence readings through the duration of the experiment, the constant temperature *C*. *vulgaris* condition was the only condition to have a significantly impeded PSII response (*F* = 87.09, *P* < 0.001). Between the two cycled temperature conditions, Chlorophyta-Ant had a significantly more responsive PSII than *C*. *vulgaris* (*F* = 6.26, *P* < 0.05). Carotenoid content was not directly measured but there was no significant yellowing observed for all experiments, indicative of significant carotenoid production, nor was there photobleaching.

## Discussion

The tested temperature environments reflecting spacecraft thermal loops had a measurable impact on the performance of both *C*. *vulgaris* and Chlorophyta-Ant sample types. Those trials with cycled media temperature had higher metabolic metrics than their constant-temperature counterparts. This suggests if using sub-optimal environmental temperatures, incorporation of a temperature respite, regardless of cycle period, may improve culture stability and metabolic function.

While both sample types increased in oxygen production over the course of the experiment, the cycled temperature *C*. *vulgaris* condition concluded with production close to three times greater than Chlorophyta-Ant. Two factors may have contributed to the extreme production rate increase for *C*. *vulgaris*, initial excess dissolved oxygen concentration and rapid generational acclimation. The initial dissolved oxygen concentration for *C*. *vulgaris* (0.013 mgO_2_ L^–1^) was almost two orders of magnitude less than Chlorophyta-Ant (0.653 mgO_2_ L^–1^). Inoculated *C*. *vulgaris* was sustained in and acclimated to a temperate lab environment (20°C). The time-averaged temperature of cycled *C*. *vulgaris* was 16°C, which could have elicited a low temperature response from the culture within the first day. Standard deviation of both *C*. *vulgaris* conditions suggest that there may have been some active oxygen consumption during the first few days of the trial. Öquist and Huner observed immediate reduction in photosynthetic activity through diversion of energy from the PSII to PSI when *C*. *vulgaris* cultures were moved from 27 to 5°C environment. They hypothesized this reduced PSII excitation pressure and minimized production of damaging reactive oxygen species (Öquist, 1983; Hüner et al., 1998; Morgan-Kiss et al., 2006; Sydney et al., 2013). Literature suggested that physiological acclimation to lower steady state temperatures may take a few hours to days including reduction in chlorophyll-a per cell, increased RuBisCO content, and eventual alterations to lipid components of thylakoid membrane (Davidson, 1991; Maxwell et al., 1994; Cao et al., 2016).

**FIGURE 6:**
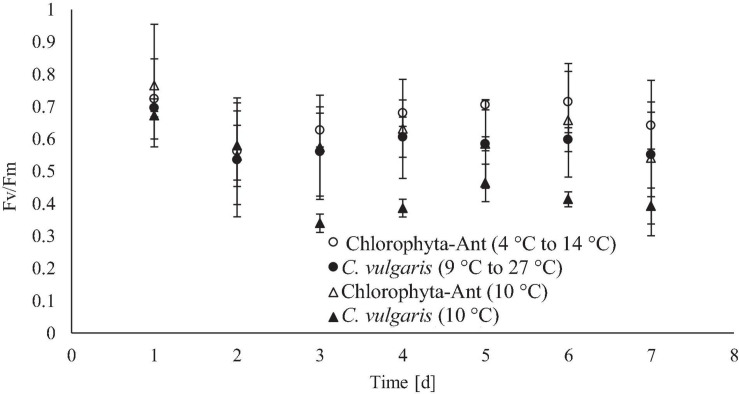
Daily maximum chlorophyll fluorescence for each tested temperature condition, measured after allowing cultures to come to a steady state temperature (19°C for *C*. *vulgaris*, 10°C for Chlorophyta-Ant).

The cycled *C*. *vulgaris* had multiple generational turnovers over the course of the experiment, with a final biomass concentration 350 times greater than the initial concentration. Morgan-Kiss et al. explained that genetic adaptation occurs over a time scale of many generations while exposed to transitory changes in environmental conditions. Therefore, it is unlikely that the culture completed genetic adaptation within these experiments, as the temperature fluctuations were within the lifetime of these cells (Morgan-Kiss et al., 2006). However, with multiple generations across the experiment duration, *C*. *vulgaris* cells were exposed to this dynamic temperature environment at infancy. This may have expedited the acclimation reflected in increased photosynthetic yield and oxygen production (Figures 4, 6). Large variations in dissolved oxygen measurements were observed during days 5, 6, and 7 for the cycled temperature *C*. *vulgaris* condition. During one experimental run, the Peltier cooler stopped working on day 4 and allowed the culture temperature to increase to 19°C for 6 h before reinitiating the temperature cycle. The experimental run continued using the same temperature cycle for the rest of day 4 and 5 through 7. The last 3 days of that isolated run resulted in substantial increases in oxygen production. The 6-h temperature respite may have increased cellular reproduction rate for a brief period, increasing biomass density and oxygen production in general.

Oxygen production normalized by the biomass dry weight elucidated any changes to oxygen production capability at the biomass level. Figure 4 illustrated increased excess dissolved oxygen for both cycled *C*. *vulgaris* and Chlorophyta-Ant. However, both *C*. *vulgaris* conditions resulted in a decrease in oxygen production per biomass over the duration of the experiment (Table 3). This suggests that the population accrued biomass, as storage products, at a disproportionate rate to oxygen production and that the culture may have been acclimating to the lower temperature regime of the tested cycle. Reallocation of energy from PSII to photosystem I (PSI) in the cold-stressed cells reduces light-dependent production of oxygen (Hüner et al., 1998, 2020; Melis, 2009). In the case of the cycled temperature *C*. *vulgaris*, the higher temperature respite periods allowed cells to increase cellular reproduction, diverting energy from protein and lipid accumulation (Yang et al., 2016). No to minimal decrease in normalized oxygen production was recorded in either Chlorophyta-Ant condition (Table 3). This suggests complete genetic adaptation of these sample types to the tested temperature environment. Sustained or increased normalized oxygen production may indicate pre-established levels of regulatory enzymes and chlorophyll for intercellular energy balance (Hüner et al., 1998, 2020). While the two different cycled temperature profiles were reflective of the LTL and MTL on the ISS and considered the optimal growing regimes of each sample type, in the future, a more balanced comparison could be made by executing the same temperature profile for both sample types.

Due to long-duration exposure to the polar environment, it was assumed Chlorophyta-Ant adaptations were well-established. These included increased polyunsaturated fatty acid concentrations within the thylakoid to sustain membrane fluidity (Morgan-Kiss et al., 2006, 2008). Cao et al. also observed that Antarctic *Chlorella* reduced the light harvesting complex (LHC) through decreased Chl-b content at low temperatures <15°C, causing reduced light absorption to prevent photoinhibition. Sustained and increased oxygen production rates in both Chlorophyta-Ant conditions suggested adaptation to the tested conditions. The equipment schedule limited the test duration to 7 days, however, increasing the duration to 14 days or a few months would have been a better reflection of long duration surface operations, and may have given more insight to the long-term acclimation of these cultures.

Microalgae will preferentially absorb carbonic acid typically available at a lower media pH (Girard et al., 2008). If negligible changes in alkalinity are assumed, increases in media pH can be associated with depletion of carbonic acid and the transition to bicarbonate consumption (Wolf-Gladrow et al., 2007). As dissolved carbon dioxide (carbonic acid in pH ≤ 6) was fixed by the culture, the pH started to increase (Figure 6). Additionally, the microalgae released hydroxide ions corresponding with photosynthetic activity, which further increased the media pH. Mayo (1997) suggested that the optimal pH for *C*. *vulgaris* and other freshwater microalgae was between 4.0 and 8.5 (Scherholz and Curtis, 2013). Both cycled temperature conditions were beyond the optimal media pH by day six of the trials. Long-term operation of photobioreactors may require scheduled sparged carbon dioxide impulses with bicarbonate dosing and biomass removal to keep the system within a favorable pH range. The experiments for this study were conducted at 84.1 kPa with approximately 0.04% CO_2_ (0.034 kPa), which is approximately a fifteenth of the carbon dioxide concentration on the ISS (0.507 kPa) (Anderson et al., 2018). However, terrestrial studies with temperate *Chlorella* have demonstrated positive correlation of photosynthetic rate with influent carbon dioxide concentration, up to 6% (v/v) (Chinnasamy et al., 2009). The variability in initial biomass concentration and culture productivity contributed to the wide standard deviation in pH measurements. It was difficult to homogenize the Chlorophyta-Ant samples for precise initial biomass densities, which led to some variability in initial biomass densities. However, the standard deviation in the pH measurements is constant across these tests. The standard deviation for pH measurements of the cycled temperature *C*. *vulgaris* condition increased on day four, in conjunction with the culture temperature coming to a constant 19°C for 6 h. This increased carbon dioxide fixation by the culture, which dramatically increased the pH measurements for this test and increased the condition’s standard deviation. On day seven, the standard deviation decreased for this condition, as the other tests increased their pH, as expected.

### Culture Volume for Surface Habitat Support

The launch mass of supply missions may constrain surface habitat infrastructure, thereby directing systems engineers to design for habitat system efficiency. If using the culture density prepared for these experiments, three times as much volume of Chlorophyta-Ant than *C*. *vulgaris* (but only two times as much biomass) is needed for sufficient oxygen supply. These estimated magnitudes in volume and dry weights are reflective of the tested environment (irradiance and CO_2_ concentration). Photobioreactors designed and optimized for oxygen production in spaceflight applications estimate needing approximately 20–200 L of culture per crewmember (Javanmardian and Palsson, 1992). Testing the temperature profiles presented here with greater biomass density cultures, to investigate density limitations, may help reduce the required culture volume for oxygen provision. However, increasing the culture density may reduce irradiance penetration length, change mixing patterns and nutrient availability, thereby possibly changing the oxygen production rate.

### Modifying Irradiance for Culture Stress Reduction

Culture health and stress levels were estimated by chlorophyll fluorescence measurements using maximum quantum yield (F_v_/F_M_). Ritchie (2006) explains that nominal measurements for higher plants is approximately 0.7–0.83, however, microalgae may have lower values closer to 0.65–0.8, with values <0.6 associated with stress (Masojídek et al., 2004; Schreiber, 2004; Ritchie, 2006). *C*. *vulgaris* at a constant 10°C was the only series that was <0.6 for more than two consecutive days, signifying suboptimal PSII response. These measurements detected photoinhibition and loss of function of the reaction centers through reduction in F_v_/F_M_ (Guidi et al., 2019). Maxwell et al. (1994) also observed reduced F_v_/F_m_ measurements (approximately 0.55) for *C*. *vulgaris*, attributed this to excess excitation pressure on PSII at the tested temperature and irradiance (constant 5°C and 150 m^–1^ s^–1^) (Vonshak et al., 2001; Yang et al., 2016).

The experiments in this study used 24-h low-level irradiance (120 μmol m^–1^ s^–1^), which may have incited photoinhibition of *C*. *vulgaris*. Increasing the environmental temperature or reducing the irradiance promotes cellular equilibrium and increases maximum quantum yield. Öquist (1983) compared the inhibition of electron transport for higher plants and noted that there was less photoinhibition when plants chilled in darkness than in high irradiance. They also stated this chilling stress was observed in thermophilic cyanobacteria but was recoverable (hours to days) after returning to optimal temperatures (Öquist, 1983; Ensminger et al., 2004; Hüner et al., 2020). While the experiments presented here tested culture performance under 24-h irradiance, it may not be reflective of potential system operation. Depending on the heat exchanger interface or photobioreactor installation in the crewed habitat, these cultures may experience periods of darkness. Recognizing that cooling periods would typically be associated with darkness (culture flowing into a metal heat exchanger or night of a Martian sol) may relieve some of the PSII excitation pressure. Conversely, increasing irradiance would nominally be tied to warming periods (habitat-installed photobioreactor exposed to environmental lighting or day of a Martian sol). Coupling environmental temperature and photoperiods may result in enhance culture performance in the cooling loop thermal regime. In the MDV, the algal mats from which the Chlorophyta-Ant culture is derived, are growing under a daily cycle of cooler stream temperatures (4°C) when the sun is low on the horizon and warmer temperatures (up to 15°C) when the sun is directly overhead, and the light intensity is greatest. Adaptation to these conditions may be a factor contributing to the Chlorophyta-Ant performing better in normalized oxygen production and photosynthetic yield.

### Impact of Growth Rate on System Stability

Cycled temperature Chlorophyta-Ant had a significantly lower biomass yield rate than its *C*. *vulgaris* counterpart (Table 2). Field studies have documented gradual growth, as these green mats are slow to reestablish after scouring by major flood events (Kohler et al., 2015). Increasing time between harvest by using slow-growing microalgae may be beneficial for specific types of system operation. Biomass harvesting and processing will require added energy with filters and pumps or crew time. Proposed Martian mission plans include dormant periods between crew occupations (Williams-Byrd et al., 2016; Badger et al., 2018). Reducing harvest frequency resulting from slower growth (Chlorophyta-Ant) may reduce overall energy consumption, and the potential for wasted biomass between occupied periods. Conversely, the resulting higher yield rate for cycled temperature *C*. *vulgaris* may support habitat operations that require rapid reestablishment or growth (e.g., post culture crash, oxygen or nutritional supplementation, or increase in habitat population). Biomass yield rate was calculated here due to the reduced sampling schedule. More frequent (daily) sampling could capture the onset of the exponential phase and allowing specific growth rate calculations. Thereby, enabling doubling rate calculations for harvesting rate estimation and comparison of metabolic response to environmental conditions.

Closing the carbon loop has been a focus of NASA as missions extend farther from Earth and are unable to rely on frequent resupply missions (NASA, 2015). Using harvested algal biomass as a nutritional supplement or feedstock is one way to provide loop closure. Algal biomass is touted as a superfood with a high edible biomass ratio, and high in protein and fatty acids (Kovač et al., 2013). While a majority of algal biomass is safe for human consumption, literature does not provide a clear consensus on the recommended daily amount of biomass (Hedenskog et al., 1969; Gitelson, 1992; Salisbury et al., 1997; Carlson, 2011; Caporgno and Mathys, 2018). If the biomass is not consumed or used, it will become waste and reduce the closure efficiency of the system. Preliminary investigation into the composition of the selected Antarctic algal mat identified *Prasiola*, *Chlorococcum*, and *Chlorella* (Van Horn et al., 2016). However, Van Horn et al. suggest conducting a targeted quantitative estimation of each of the identified genera for abundance mapping. This may also benefit spaceflight applications, as various dominant genera may require different preparation for human consumption (Sathasivam et al., 2019).

### Establishing Sustained Operations and Forward Work

The study presented here focused on the metabolic response of microalgal cultures to a dynamic thermal environment reflective of a spacecraft thermal loop. This study did not take into consideration the concept of operations for transporting, establishing, or sustaining these cultures. The following sections are initial suggestions for these operations based on terrestrial findings but should be further tested to establish their suitability.

A surface habitat could integrate a multifunctional bioregenerative life support system into the cabin in a multitude of ways. This study hypothesized that an algal culture would be inoculated into a water-based thermal control system mounted onto the cabin walls. The thermal control system would use opaque tubing to allow the transfer of cabin illumination and cabin heat. Pumps would keep the media constantly moving, lifting the culture, and reducing temperature and nutrient gradients. Gas transfer would be accomplished by nonporous membranes, allowing for simultaneous carbon dioxide, oxygen, and heat transfer as described in Matula and Nabity (2021). Interface heat exchangers incorporated into the loops would transfer collected heat from the media to external ammonia loops or radiator systems. Sample ports throughout the system would allow for harvesting of biomass and nutrient dosing.

It may not be prudent to transport liters of media due to volume and mass constraints, but microalgae may be transported by streaking concentrated cells on agar slants for short durations (approximately 6 months) at room temperature (Hu et al., 2020). However, this approach may require refrigeration to reduce extraneous bacterial growth. Antarctic algal mats are naturally cryodesiccated between austral summers, also reducing the amount of water for transport (Howard-Williams et al., 1986; Hawes et al., 1992). Currently, there is no consensus on proper technique for laboratory cryodesiccation of Antarctic green algal mat with high post-wetting viability percentages (Davey, 1989; Day et al., 1997; Šabacká and Elster, 2006). The Martian surface may provide water from ice harvested from polar icecaps or subglacial brine lakes for rehydration or inoculation of these cultures (O’Callaghan, 2020, 2021; Lauro et al., 2021; Scheller et al., 2021).

A slip stream of 95% CO_2_ Martian atmosphere could be collected, diluted with N_2_ or any other inert gas, and sparged into the photobioreactor system (Jha et al., 2006). Lunar surface habitats may use combustion or decomposition of harvested biomass as an additional supply of carbon dioxide. Planetary surface habitats with some amount of gravity may use the buoyancy effects of sparging system to supply carbon dioxide, release oxygen, and mix the system. Conducting experiments with Antarctic Chlorophyta exposed to elevated concentrations of carbon dioxide could help bioregenerative ECLS designers understand operational conditions but also help polar phycologists understand potential future impacts of variations in alkalinity due to accelerated weathering and increasing carbon dioxide concentrations on Antarctic algal mats.

Nutrient dosing was not studied here but it is understood that long-term operation of these photobioreactors, depending on the selected species, may need supplementation of nitrogen, phosphorous, and certain micronutrients for cell sustainment. Human urine has been considered for nitrogen and phosphorous supplementation in spaceflight applications (total nitrogen: approximately 5,600 mg L^–1^, total phosphorous: 310 mg L^–1^) (Blüm et al., 1994; Tikhomirov et al., 2007; Tuantet et al., 2014a; Pickett et al., 2020). Terrestrially, wastewater remediation has successfully cultivated microalgae (specifically *Chlorella* sp.) on wastewater (Wang et al., 2010; Jaatinen et al., 2015; Tuantet et al., 2019). Typically, these studies use diluted urine (1:2–1:200). Urea hydrolysis can rapidly occur in undiluted urine, increasing pH, and precipitating necessary nutrients, thereby reducing growth (Zhang et al., 2014).

Irradiance may be a deciding factor for efficiency of the overall air revitalization and thermal control system. Using in-situ irradiance at the planetary surface may be the most power, mass, and thermally effective way to illuminate the cultures. The Martian surface receives a photosynthetic photon flux density (PPFD) of approximately 80–350 μmol m^–1^ s^–1^ with a yearly average of 230 μmol m^–1^ s^–1^, based upon the Viking Lander 1 location (Clawson and Hoehn, 2005). The Lunar surface receives a nearly continuous 62.1 μmol m^–1^ s^–1^ at the south pole and a maximum of 2,300 μmol m^–1^ s^–1^ during its 29-day lunar day cycle (Badescu, 2012). Lighting systems using LEDs tuned to the photosynthetically active radiation (PAR) spectrum may supplement this *in-situ* resource. However, resulting heat loads require investigation of several types of irradiance sources to benefit the overall efficiency of the photobioreactor system.

Various spaceflight experiments have experienced biofilm formation, and for microalgae, this occurred in areas of concentrated irradiation (Kim et al., 2013; Zea et al., 2018; Helisch et al., 2020). Reduction in irradiance penetration length can result in systems unable to accommodate biofilm accumulation on surfaces, thereby reducing oxygen production capacity of the system (Lee and Palsson, 1994; Tuantet et al., 2014b; Helisch et al., 2020). A cleaning or flow rate regimen in conjunction with material selection may mitigate buildup. Fortunately, wall cleanliness may have minimal impact on the heat transfer capabilities of the system. Algal cells are approximately 99% water by weight (Mata et al., 2012). Gryta (2008) observed that biological deposits on heat transfer surfaces with high water content had significantly less influence on heat transfer when compared to inorganic aggregation. Therefore, if biofilming progressed, air revitalization capabilities may be affected before heat transfer capabilities.

## Conclusion

In this study, the response of *C*. *vulgaris* and Antarctic Chlorophyta sampled from the MDV to rapid temperature cycles reflective of a spacecraft thermal loop was compared. The measured oxygen production and yield rates were used to estimate the feasibility of using these microalgae for simultaneous closed-loop air revitalization and thermal control in a surface habitat. Due to the intrinsic psychrotolerant properties of Antarctic Chlorophyta, it was hypothesized that these microalgae would outperform their temperate *C*. *vulgaris* counterparts in the tested temperature regimes. Holding the environmental temperature of *C*. *vulgaris* at a constant 10°C significantly reduced culture growth and oxygen production, and stressed culture PSII. However, cycling temperature between +9 and +27°C provided environmental respite, reduced cellular stress, and promoted environmental acclimation, observed through increasing media pH, chlorophyll fluorescence, and excess dissolved oxygen concentration. The tested temperature regimes elicited minimal stress from both Antarctic Chlorophyta conditions, as normalized oxygen production either increased or had a minimal decrease and chlorophyll fluorescence was greater in this sample type than in *C*. *vulgaris*. However, even with the temperature-induced reduction, cycled temperature *C*. *vulgaris* had a significantly higher normalized oxygen dissolved oxygen concentration than Antarctic Chlorophyta. This preliminary investigation suggests that both *C*. *vulgaris* and Antarctic Chlorophyta can withstand the dynamic temperature environment reflective of a thermal control loop. Using a bioregenerative approach to simultaneous air revitalization and thermal control may provide carbon loop closure for long-duration surface habitats. However, mission design requirements including radiation environment, habitat thermal loads, and air revitalization demands may favor one tested sample type over the other. Therefore, future research should include longer-duration trial runs with both sample types using the same temperature profile for a balanced comparison. Including elevated carbon dioxide environments and variation in irradiance regimes may also be a better reflection of the surface habitat environment.

## Data Availability Statement

The raw data supporting the conclusions of this article will be made available by the authors, without undue reservation.

## Author Contributions

EM developed the concept and design of the study, executed the experiments, produced the statistical analysis, and wrote the first draft of the manuscript. All authors contributed to manuscript revision, read, and approved the submitted version.

## Conflict of Interest

The authors declare that the research was conducted in the absence of any commercial or financial relationships that could be construed as a potential conflict of interest.

## Publisher’s Note

All claims expressed in this article are solely those of the authors and do not necessarily represent those of their affiliated organizations, or those of the publisher, the editors and the reviewers. Any product that may be evaluated in this article, or claim that may be made by its manufacturer, is not guaranteed or endorsed by the publisher.

## Abbreviations

Chl-a,b: chlorophyll-a,b
CO_2_: carbon dioxide
ECLS: environmental control and life support
F_v_/F_M_: maximum fluorescence
IATC: internal active thermal control
ISS: International Space Station
*k_*stir*_a*: volumetric mass transfer coefficient (d^–1^)
LHC: light harvesting complex
LTL: lower temperature loop
LEO: low earth orbit
MDV: McMurdo Dry Valleys
MTL: moderate temperature loop
*N*_0,t_: biomass dry weight at time *t*
O_2_: oxygen
O^∗^_2sat_: dissolved oxygen saturation in water at measured temperature (g L^–1^)
O_2t_: dissolved oxygen at time *t*
Δ O_2_: excess dissolved oxygen (g L^–1^)
PAM: pulse amplitude modulation
PS I,II, photosystem I: II
Y: biomass yield rate (gDW L^–1^d ^−1^).
